# Noncoding RNAs in Cardiac Autophagy following Myocardial Infarction

**DOI:** 10.1155/2019/8438650

**Published:** 2019-06-03

**Authors:** Annie Turkieh, Henri Charrier, Emilie Dubois-Deruy, Sina Porouchani, Marion Bouvet, Florence Pinet

**Affiliations:** Univ. Lille, Inserm, Institut Pasteur de Lille, U1167-RID-AGE, FHU-REMOD-VHF, F-59000 Lille, France

## Abstract

Macroautophagy is an evolutionarily conserved process of the lysosome-dependent degradation of damaged proteins and organelles and plays an important role in cellular homeostasis. Macroautophagy is upregulated after myocardial infarction (MI) and seems to be detrimental during reperfusion and protective during left ventricle remodeling. Identifying new regulators of cardiac autophagy may help to maintain the activity of this process and protect the heart from MI effects. Recently, it was shown that noncoding RNAs (microRNAs and long noncoding RNAs) are involved in autophagy regulation in different cell types including cardiac cells. In this review, we summarized the role of macroautophagy in the heart following MI and we focused on the noncoding RNAs and their targeted genes reported to regulate autophagy in the heart under these pathological conditions.

## 1. Introduction

Myocardial infarction (MI) is a cardiovascular event caused by obstruction of one or more arteries supplying the heart. This area of the heart is therefore no longer supplied with oxygen and nutrients leading to the death of cardiomyocytes. Coronary reperfusion is the only recognized method to reduce the size of the infarct if it is performed within hours after MI. Despite its beneficial effect, several deleterious events such as increased oxidative stress and cell death are observed during the reperfusion process. If the infarcted zone is very extensive, there is a decrease in the contractile function of the heart. In order to compensate for this loss and maintain normal blood flow, the heart will undergo structural changes such as thinning of the infarcted zone, fibrosis, cardiomyocyte hypertrophy, and left ventricle (LV) dilatation [1]. Left ventricle remodeling (LVR) is initially a protective mechanism but in the long term can lead to heart failure (HF) [2–4]. Despite current therapy, acute MI and HF remain the leading causes of death and disability worldwide. New therapeutic strategies are therefore required to protect the heart against the detrimental effects of acute ischemia/reperfusion (I/R) injury, in order to prevent cardiomyocyte death and reduce myocardial infarct size, preserve LV function, and prevent the onset of HF.

Macroautophagy is an important and nonselective proteolytic mechanism that regulates the homeostasis of long-lived proteins, macromolecules including lipids and cell organelles, by surrounding them in a double-membrane vesicle known as autophagosome in order to deliver them to the lysosome for degradation [5]. It plays an essential role for maintaining heart structure and function under baseline conditions [6–8]. Several studies showed that macroautophagy is upregulated in the heart following MI and suggested that this process may protect the heart against MI effects [9–11]. Recently, it was shown that noncoding RNAs (microRNAs (miRNA) and long noncoding RNAs (lncRNA)) are involved in autophagy regulation in different cell types including cardiac cells [12–14]. In this review, we summarized the role of macroautophagy in the heart following MI and we focused on the noncoding RNAs and their targeted genes reported to regulate autophagy in the heart under pathological conditions.

## 2. Macroautophagy Mechanism

Macroautophagy proceeds in several successive steps and involves different proteins as previously described [5]. In summary, autophagy induction is mainly regulated by the ULK (unc-51-like kinase) complex which is composed of ULK1/2, ATG13 (autophagy-related gene 13), ATG101, and FIP200 (focal adhesion kinase family interacting protein with a 200 kDa mass). Activation of the PI3K complex contributes to the vesicle nucleation, the first step of autophagosome formation. This complex is composed of Beclin-1, ATG14, VPS34 (phosphatidylinositol 3-kinase vacuolar protein sorting 34), and VPS15. Finally, two ubiquitin-like protein conjugation systems are required for the vesicle elongation, the first to form ATG12-ATG5-ATG16L1 complex and the second to form LC3II (microtubule-associated protein 1 light chain II), the lipidated form of LC3. For this latter step, ATG4 cleaves pro-LC3 to LC3I before its conjugation to phosphatidylethanolamine by ATG7, ATG3, and ATG12-ATG5-ATG16L1 complex. Several pathways were shown to regulate autophagy by activation or inactivation of one of these ATG proteins. For example, mTOR (mammalian target of rapamycin) activation inhibited autophagy by decreasing ULK1 activity [15] and ATG14/VSP34-35 complex formation [16]. AMPK (adenosine monophosphate-activated protein kinase) positively regulated autophagy by increasing Beclin-1 phosphorylation leading to its interaction with VSP34 [17]. However, Bcl-2 interacts with Beclin-1 for blocking its interaction with VSP34 [18].

### 2.1. Macroautophagy during Ischemia/Reperfusion

The regulation of autophagy is different during ischemia and reperfusion [10]. During heart ischemia, nutrient and oxygen supplies to the cardiac cells decrease, inducing mitochondrial and cellular dysfunction that lead to cell death. To protect them, the cardiac cells induce autophagy via the AMPK/m-TOR pathway in order to degrade/eliminate damaged organelles and proteins and provide the substrates necessary for their survival. During reperfusion, there is an increase of reactive oxygen species (ROS) production inducing a strong expression of Beclin-1 which on the one hand promotes the formation of autophagosomes and on the other hand inhibits the expression of genes involved in the fusion of autophagosomes with lysosomes [19]. In addition, ROS inhibit the expression of LAMP-2, a protein involved in the fusion of autophagosomes with lysosomes. Autophagy is then induced excessively during reperfusion but is inactive. Blocking the degradation of the contents of autophagosomes promotes oxidative stress, decreases mitochondrial permeability, and causes cell death. Partial inhibition of Beclin-1 expression (heterozygous mice) has been shown to protect against apoptosis induced during reperfusion while its total deletion is deleterious [10]. These data showed that autophagy is a protective mechanism during ischemia but its excessive induction during reperfusion is deleterious.

### 2.2. Macroautophagy during LVR in Post-MI

The activity of autophagy and its role in LVR post-MI have been studied in murine models with permanent ligation of the left coronary artery. Autophagy is induced in noninfarcted area of the heart during the subacute (1 week) and chronic (3 weeks) stages after MI [11]. Inhibition of autophagy by bafilomycin (a pharmacological agent that blocks the fusion of the autophagosome with the lysosome) promoted LVR and worsens cardiac dysfunction. In contrast, administration of trehalose (a nonnaturally reduced disaccharide) in mice after ligation activated autophagy, reduced LVR, and improved cardiac function at 4 weeks post-MI [20]. However, this protective effect of trehalose on the heart was not observed in mice invalidated for the Beclin-1 gene, but an increase in the activity of mTOR was observed in the noninfarcted area of the heart. It has been shown that the inhibition of mTOR activity induced autophagy leading to a decrease of LVR and an improvement in cardiac function in post-MI [21]. All these data showed a protective role of autophagy in later stages in post-MI but its activity remained insufficient to prevent LVR and cardiac dysfunction.

## 3. Macroautophagy Regulation by Noncoding RNAs during and following MI

About 99% of the human genome do not encode proteins but are transcriptionally highly active and give rise to a broad spectrum of noncoding RNAs (ncRNAs) with regulatory and structural functions. Based on the size criteria of 200 nucleotides (nt), ncRNAs are divided into long (>200 nt) and short ncRNAs (<200 nt).

**Figure 1:**
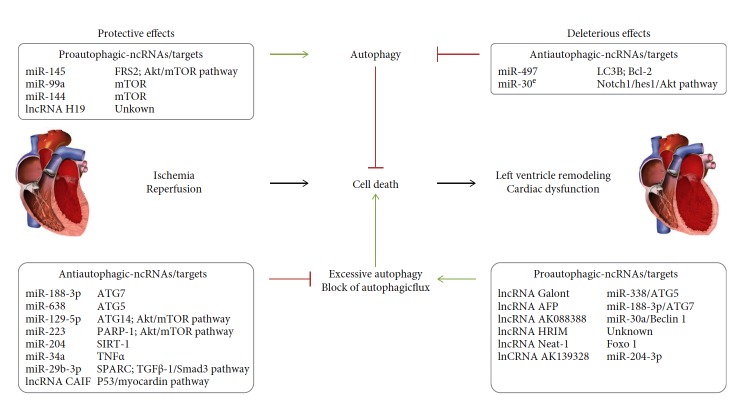
Outline summarizing the noncoding RNAs regulating cardiac autophagy and their targets and function. Green and red arrows indicate activation and inhibition, respectively.

The ncRNAs are modulated in some cardiovascular diseases including MI [22, 23]. The significant changes in their expression pattern upon MI highlighted their contribution in the regulation of pathogenesis of MI. Furthermore, it was shown that ncRNAs could regulate autophagy in some cardiac disorders including MI, hypertrophy, and HF [12–14]. In this part, we summarized the noncoding RNAs which have been reported to regulate cardiac autophagy during and following MI and highlighted their specific autophagic targets and their importance as new therapeutic targets to protect the heart against I/R injury and prevent cardiac remodeling and dysfunction (Figure 1).

### 3.1. Macroautophagy Regulation by mRNAs

MiRNAs are defined as single-stranded noncoding RNAs around 22 nucleotides and are highly conserved between species [22]. Once synthetized and matured through several steps, these miRNAs bind to the complementary 3′UTR of their target mRNA and either degrade or silence them. A near-perfect match between the seed region of the miRNA (8 nucleotides at its 5′UTR end) and its target leads to complete degradation of mRNA, while a partial complementary results in the suppression of the gene expression. MiRNAs may have one or multiple mRNA targets and are involved in the regulation of numerous biological processes in the heart including autophagy.

#### 3.1.1. Antiautophagic miRNAs with Protective Effects

Several miRNAs were modulated during I/R and seem to have a protective effect by decreasing excessive autophagy-induced cell apoptosis by targeting one of the ATG genes. MiR-188-3p levels are reduced in cardiomyocytes treated with anoxia/reoxygenation and in MI mice. Overexpression of miR-188-3p in MI mice attenuated autophagy by targeting autophagy mediator Atg7 and decreased the infarcted area size [24]. It was shown that miR-638 suppressed the expression of Atg5 by targeting its 3′UTR region. It is downregulated in human cardiomyocytes after hypoxia/reoxygenation (H/R), and its overexpression improves the viability of these cells. However, enforced expression of Atg5 reversed the effect of miR-638 on autophagy and cell apoptosis suggesting that miR-638 attenuated the effects of H/R treatment by regulating ATG5-mediated autophagy in human cardiomyocytes [25]. Also, overexpression of miR-129-5p in H9c2 cells treated by hydrogen peroxide inhibited autophagy by targeting the Atg14 gene and activating the PI3K/AKT/mTOR pathway resulting in decreased cell apoptosis [26].

Other miRNAs play their protective effect by regulating one of the pathways involved in autophagy regulation. The levels of miR-223 are significantly upregulated in the heart of post-MI HF rats and in hypoxia-treated neonatal rat cardiomyocytes (NRCMs) and H9c2 cells. The increased miR-223 levels protect NRCMs and H9c2 cells from hypoxia-induced apoptosis whereas decreasing miR-223 expression had contrasting effects. This protective effect of miR-223 is explained by the decrease of its target gene expression PARP-1 (poly(ADP-ribose) polymerase 1) resulting in inhibition of excessive autophagy via the Akt/mTOR pathway [27]. However, miR-204 expression is decreased in the heart of rat upon I/R injury associated with increased autophagy as observed by the increased LC3II levels [28]. Also, it was shown that transfection of miR-204 in H9c2 cells attenuated cell apoptosis induced by H/R treatment. The protective effect of miR-204 is explained by targeting SIRT1-mediated autophagy [29]. The expression of miR-34a is also decreased during I/R and overexpression of this miR decreased TNF*α* expression resulting in reduced autophagy and apoptosis levels on NRCMs after H/R [30]. Lower miR-29b-3p levels were found in HF patients and in hypoxia-stimulated H9c2 cells. The overexpression of miR-29b-3p inhibited autophagy and apoptosis induced in hypoxic-induced H9c2 cells through targeting SPARC and inhibiting TGF*β*-1/Smad3 pathway [31].

#### 3.1.2. Antiautophagic miRNAs with Deleterious Effects

Some miRNAs contribute to ischemic/reperfusion injury by inhibiting the autophagy process. miR-497 is dramatically downregulated in the infarcted heart and in hypoxic cardiomyocytes, and its overexpression in murine MI model increased the infarcted size. It was shown that miR-497 inhibited autophagy by targeting the LC3B gene and induced cell apoptosis by targeting the Bcl-2 gene suggesting that decreasing miR-497 levels is a protective mechanism of the heart in response to MI [32]. The expression of miR-30e was also decreased after myocardial I/R. Its silencing in H9c2 cells increased autophagy and attenuated oxidative stress and cell apoptosis that are reversed by treating the cells with 3-methyladenine, an inhibitor of macroautophagy. These results suggest that decreasing the miR-30e levels protected the heart against I/R injury by autophagy induction [33].

#### 3.1.3. Proautophagic miRNAs with Protective Effects

Higashi et al. [34] showed that 30 min of coronary occlusion followed by 2 days of reperfusion caused a significant decrease in the rabbit cardiac tissue expression of miR-145 in the border and infarcted areas of the myocardium compared to the remote noninfarcted area. Injection of liposomes containing miR-145 after the beginning of reperfusion reduced the infarcted area size and improved the LV function and remodeling; these beneficial effects were abolished by chloroquine treatment. Further study showed that miR-145 promoted autophagy in cardiomyocyte by directly targeting FRS2 (fibroblast growth factor receptor substrate 2) mRNA resulting in the acceleration of the transition of LC3I to LC3II, an important step of autophagosome maturation [34]. The protective effect of miR-145 is also observed in H9c2 cells after H/R. In this study, the authors demonstrated that miR-145 inhibited H/R-induced apoptosis by promoting autophagy via the Akt3/mTOR signaling pathway [35]. miR-99a was shown to be downregulated in the infarcted heart and in neonatal mice ventricle myocytes exposed to hypoxia. Lentivirus-mediated overexpression of miR-99a in the infarcted heart inhibited cardiac remodeling and improved heart function at 1 and 4 weeks after its administration. It was shown that miR-99a decreased mTOR protein levels without any effect on its mRNA levels suggesting that miR-99a regulated mTOR expression at a posttranscriptional level. Consequently, the autophagy induced was associated with a decrease of cell apoptosis. This study demonstrated that overexpression of miR-99a improved post-MI cardiac function by upregulating autophagy via targeting mTOR pathways, inhibiting apoptosis, and attenuating pathological remodeling [36]. The miR-144 levels were reduced in the heart of MI mice with permanent left anterior descending artery (LAD) ligation. The miR-144 k/o mice showed a worse HF phenotype with ventricular dilatation and impaired contractility after LAD ligation. However, miR-144 administration decreased myocardial infarcted size and improved post-MI remodeling. Further study allowed authors to conclude that miR-144 increased autophagy and decreased fibrosis and apoptosis by targeting mTOR [37].

### 3.2. Macroautophagy Regulation by lncRNAs

lncRNAs are noncoding RNAs longer than 200 nucleotides that regulate both gene expression and protein translation [22]. Nuclear localized lncRNAs can regulate gene expression at both the epigenetic and transcriptional levels. Cytosol-based lncRNAs can modify protein translation by blocking, stabilizing/destabilizing, or sponging miRNAs. The lncRNAs are involved in the regulation of numerous biological processes including autophagy in cardiac and noncardiac cells.

#### 3.2.1. Antiautophagic lncRNA with Protective Effects

Liu et al. [38] showed that the expression of lncRNA CAIF (cardiac autophagy inhibitory factor) was significantly decreased in a mice model of I/R injury and in cardiomyocytes treated with H_2_O_2_. Conversely, overexpression of CAIF inhibited autophagy inducing cardiomyocyte cell death and cardiac dysfunction caused by I/R. In this study, the authors demonstrate that CAIF directly binds to p53 protein and blocks its interaction with the myocardin promotor. Myocardin, a smooth muscle and cardiac muscle-specific transcriptional activator, is upregulated after I/R and H_2_O_2_ treatment and is involved in autophagy regulation in cardiomyocytes by increasing Beclin-1 expression. These data suggest CAIF-P53-myocardin pathway as a novel regulator of autophagy in cardiomyocytes and as a potential therapeutic target in order to inhibit excessive autophagy and improve cardiac function after I/R [38].

#### 3.2.2. Proautophagic lncRNA with Protective Effects

On the other hand, it was shown that the lncRNA H19 expression was decreased in a mice model of acute MI and that its overexpression decreased infarcted size and improved cardiac function associated with autophagy upregulation; however, the mechanisms by which autophagy is regulated by H19 are still unknown. These results suggest that H19 protects the heart from MI by increasing cardiac autophagy [39].

#### 3.2.3. Proautophagic lncRNAs with Deleterious Effects

Some lncRNAs are upregulated after I/H and enhanced autophagy target gene expression by inhibiting miRNA expression. Yin et al. [40] showed that lncRNA Galont (GATA1 activated lncRNA) is upregulated in neonatal mice cardiomyocytes in response to anoxia/reoxygenation; however, miR-338 expression is downregulated. Overexpression of miR-338 directly decreased the formation of autophagic vesicles and induced cell death after anoxia/reoxygenation treatment without any effect on control cells. The antiautophagic effect of miR-338 is explained by its direct targeting of the autophagic mediator Atg5. It was shown that Galont directly bound to miR-338 and decrease its expression. Consequently, Atg5 expression is increased resulting in excessive cardiac autophagy and cell death [40]. Also, the lncRNA APF (autophagy-promoting factor) enhances cardiac autophagy and cell death by inhibiting miR-188-3p expression resulting in the increase of its target gene expression, Atg7 [24]. Furthermore, lncRNA AK088388 is upregulated during reoxygenation in mouse cardiac myocytes associated with the decreased miR-30a expression. Overexpression of miR-30a decreased the expression of its target gene Beclin-1 resulting in inhibition of autophagy induction and decreased cell death. The cooverexpression of lncRNA AK088388 inhibited the protective effect of miR-30a. However, the mutation of the miR30-a binding site in AK088388 failed to block the effect of this miRNA on autophagy and cell survival. These results suggest that lncRNA AK088388 regulates autophagy through miR-30a/Beclin-1 pathway to affect cardiomyocyte injury [41]. The lncRNA HRIM (hypoxia/reoxygenation injury-related factor in myocyte) was upregulated after H/R in H9c2 cells. HRIM silencing prevented death of cells by suppressing the autophagic activity in H/R-treated cells. However, the target genes of this lncRNA and the detailed mechanism of its autophagic effect need to be elucidated [42]. Other lncRNAs were highly expressed in diabetic murine heart and contributed to I/R injury by regulating autophagy. It was shown that Neat-1 (nuclear-enriched abundant transcript 1) and AK139328 seemed to induce autophagy by upregulating Foxo1 expression and decreasing miR-204-3p levels, respectively [43, 44].

lncRNA MALAT1 (metastasis-associated lung adenocarcinoma transcript 1) is expressed at high levels in patients with acute MI [45] and is closely associated with the pathogenesis of myocardial I/R injury [46, 47]. It was shown, on the one hand, that MALAT1 contained binding site for miR-204 [48] and, in the other hand, that miR-204 protected the cardiomyocytes against I/R injury via inhibiting autophagic cell death [28]. Also, MALAT1 targeted miR-558 to enhance ULK1-mediated autophagy in isoproterenol-treated cardiomyocytes [49]. It will be important to know if lncRNA MALAT increased cardiomyocyte autophagy and myocardial injury during I/R by negatively regulating miR-204 or miR-558 expression.

## 4. Conclusion

Despite current therapies, acute MI and HF which often follows remain the leading causes of death and disability worldwide. New therapeutic strategies are therefore required to protect the heart against the detrimental effects of acute ischemia/reperfusion injury. Inhibition of macroautophagy during reperfusion prevented cardiomyocyte death and reduced myocardial infarct size; however, its induction during LVR preserved LV function and prevented the onset of HF. Most pharmacological agents used up to date for regulating macroautophagy are not specific and may interfere with other cellular processes, so it will be necessary to identify new therapeutic approaches to regulate autophagy. Several noncoding RNAs were shown to be modulated during I/R and involved on cardiac autophagy regulation. The tissue-specific expression of some noncoding RNAs and their easy manipulation show their potential as novel targets for clinical developments to treat autophagy-related diseases. Identification of specific cardiac noncoding RNAs that regulate autophagy could be a good opportunity to protect the heart from MI injury without affecting the autophagy activity in other organs.
